# Peroxisome Proliferator-Activated Receptor γ Deficiency in T Cells Accelerates Chronic Rejection by Influencing the Differentiation of CD4+ T Cells and Alternatively Activated Macrophages

**DOI:** 10.1371/journal.pone.0112953

**Published:** 2014-11-10

**Authors:** Xiaofan Huang, Lingyun Ren, Ping Ye, Chao Cheng, Jie Wu, Sihua Wang, Yuan Sun, Zheng Liu, Aini Xie, Jiahong Xia

**Affiliations:** 1 Department of Cardiovascular Surgery, Union Hospital, Tongji Medical College, Huazhong University of Science and Technology, Wuhan, People’s Republic of China; 2 Department of Cardiology, Central Hospital of Wuhan, Wuhan, People’s Republic of China; 3 Department of Thoracic Surgery, Union Hospital, Tongji Medical College, Huazhong University of Science and Technology, Wuhan, People’s Republic of China; 4 Division of Diabetes, Endocrinology and Metabolism, Department of Medicine, Baylor College of Medicine, Houston, Texas, United States of America; 5 Department of Cardiovascular Surgery, Central Hospital of Wuhan, Wuhan, People’s Republic of China; Virginia Tech, United States of America

## Abstract

**Background:**

In a previous study, activation of the peroxisome proliferator–activated receptor γ (PPARγ) inhibited chronic cardiac rejection. However, because of the complexity of chronic rejection and the fact that PPARγ is widely expressed in immune cells, the mechanism of the PPARγ - induced protective effect was unclear.

**Materials and Methods:**

A chronic rejection model was established using B6.C-H-2^bm12^KhEg (H-2^bm12^) mice as donors, and MHC II-mismatched T-cell-specific PPARγ knockout mice or wild type (WT) littermates as recipients. The allograft lesion was assessed by histology and immunohistochemistry. T cells infiltrates in the allograft were isolated, and cytokines and subpopulations were detected using cytokine arrays and flow cytometry. Transcription levels in the allograft were measured by RT-PCR. In vitro, the T cell subset differentiation was investigated after culture in various polarizing conditions. PPARγ-deficient regularory T cells (Treg) were cocultured with monocytes to test their ability to induce alternatively activated macrophages (AAM).

**Results:**

T cell-specific PPARγ knockout recipients displayed reduced cardiac allograft survival and an increased degree of pathology compared with WT littermates. T cell-specific PPARγ knockout resulted in more CD4+ T cells infiltrating into the allograft and altered the Th1/Th2 and Th17/Treg ratios. The polarization of AAM was also reduced by PPARγ deficiency in T cells through the action of Th2 and Treg. PPARγ-deficient T cells eliminated the pioglitazone-induced polarization of AAM and reduced allograft survival.

**Conclusions:**

PPARγ-deficient T cells influenced the T cell subset and AAM polarization in chronic allograft rejection. The mechanism of PPARγ activation in transplantation tolerance could yield a novel treatment without side effects.

## Introduction

In end-stage heart disease, heart transplantation is becoming the most important clinical treatment [1]. However, even with efficient immunosuppressive therapy to prevent acute rejection in the early stage after transplantation, chronic allograft rejection is the major obstacle to the long-term survival of heart transplant recipients [2]. The principal phenomena causing chronic allograft rejection are coronary allograft vasculopathy (CAV) [3] and leukocytes from recipients infiltrating into allografts [4]. Previous studies have demonstrated that an immune mechanism participates in chronic allograft rejection. Many studies have focused on CD4+ T helper cells and their subsets, such as Th1, Th2, Th17 and regulatory T cells (Treg), in the process of chronic allograft rejection. Th1 and Th17 secrete the pro-inflammatory cytokines interferon (IFN)-γ and IL-17A, which are known to promote chronic allograft rejection [5], [6]. Th2 cells secrete IL-4, IL-5, IL-10, and IL-13, whereas Treg have an immunoregulatory function that has protective effects in numerous situations [7], [8]. CD4+ T helper cells and their associated cytokines can influence the function and polarization of macrophages [9]. The classically activated macrophage (CAM)/alternatively activated macrophage (AAM) ratio in allografts has been considered to play a key role in the immune response to transplantation [10]. Within the complex mechanism of chronic allograft rejection, both T cells and macrophages participate in the lesion of allografts [3]. These cells are immunotherapy targets in chronic allograft rejection.

Peroxisome proliferator-activated receptor-γ (PPARγ) is a member of a nuclear receptor family that regulates glucose metabolism and lipogenesis. Recently, PPARγ and its agonists were found to have immunoregulatory functions in T cells and macrophages [11]–[13]. Given the anti-inflammatory effects of PPARγ agonists, we and other researchers have used them to treat both acute and chronic allograft rejection and have observed clear protective effects [14]–[16]. However, due to the complex pathological process of chronic rejection and the broad effects of PPARγ on multiple immune cells, details regarding the effects of PPARγ on immune cells in chronic allograft rejection are unclear.

To understand the mechanism of PPARγ and its agonists in chronic allograft rejection, we used B6.C-H-2^bm12^KhEg (H-2^bm12^) mice as donors and T cell specific PPARγ knockout (PPARγ fl/fl; Lck-Cre^+^, T-cell-PPARγ^ko^) mice or wild type (WT) littermates as recipients to establish a single major histocompatibility complex (MHC) class II-mismatched cardiac chronic allograft rejection model. We found that T cell-specific PPARγ deficiency impacted the differentiation of CD4+ T cell subsets and AAM bias in cardiac allografts. The protective effect of PPARγ agonists was eliminated in PPARγ deficiency in T cells.

## Materials and Methods

### Animals

B6.129-Ppargtm2Rev/J (H-2^b^, in short PPARγ fl/fl) mice, B6.Cg-Tg (Lck-cre)548Jxm/J (H-2^b^, in short Lck-Cre^+^) mice and B6.C-H-2^bm12^KhEg (H-2^bm12^, in short bm12) mice were purchased from Jackson Laboratories Inc (Bar Harbor, ME, USA). C57BL/6 (H-2^b^) mice were purchased from Tongji Medical College of Huazhong University of Science and Technology (HUST) (Wuhan, China). T cell-PPARγ^ko^ mice were generated by crossbreeding and verified by the standard PCR procedure recommended by Jackson Labs. PPARγ fl/fl; Lck-Cre^+^ T cell- PPARγ^ko^ mice and PPARγ fl/fl; Lck-Cre^−^ WT littermates were regarded as controls for each other. All of the experimental mice were male and 6 to 8 weeks old (18–25 g body weight). The animals were bred and maintained in a specific pathogen-free (SPF) barrier facility at Tongji Medical College (Wuhan, China). All animal experiments were approved by the Institutional Animal Care and Use Committee of Tongji Medical College. The protocol was approved by the Ethics Committee of Tongji Medical College, Huazhong University of Science and Technology (IORG No: IORG0003571). All surgery was performed under sodium pentobarbital anesthesia, and all efforts were made to minimize suffering.

### Cardiac transplantation model and treatment

Heterotopic vascularized cardiac transplantation models were established as described previously [17]. Donor and recipient mice were anesthetized by intraperitoneal injection with ketamine at 1 mg/kg body weight. The donor hearts were transplanted into the abdomens of the recipients; the ascending aorta was connected to the recipient abdominal aorta and the pulmonary artery was sutured with the recipient inferior vena cava. The recipient mice were maintained on a thermal place until recovery from operation. Allograft function was monitored by daily palpation. At the endpoint of experiments, mice were sacrificed by an over dose injection of ketamine and heart grafts were harvested for further detection. Pioglitazone (Ji’nan Zhongke Yitong Chemical Co., Ltd, Ji’nan, China) was provided in the chow (3 mg/kg*d) [15] starting at 1 day prior to the operation.

### Histological analyses of allografts

Allografts from recipients were harvested 40 days after the operation. The allograft was embedded in paraffin for hematoxylin and eosin (H&E) and Masson trichrome staining. The severity of each allograft rejection was scored according to graft coronary artery disease (GAD) and parenchymal rejection (PR) grade [18], [19]. A portion of the allograft was embedded in OCT (Sakura, Torrance, CA, USA) and subjected to immunohistochemical staining using anti-mouse CD4, CD8, and γδTCR antibodies (eBioscience, San Diego, CA).

### Isolation of infiltrated lymphocytes from allografts and flow cytometry

The infiltrated lymphocytes were isolated as described previously [20]. The allografts were cut into pieces and digested with 2 mg/ml collagenase D (Worthington Bio, Lakewood, NJ) combined with 10% fecal calf serum in RPMI 1640 media for 2 h at 37°C. The suspensions were filtered with cell strainers (40 µm, BD Biosciences, San Diego, CA). The isolated cells were stained with FITC-anti-CD4, APC-Cy7-anti-CD11b, PerCP-Cy5.5-anti-IFN-γ, eFlour660-anti-IL-13, PE-anti-IL-17A, APC-anti-Foxp3, and 7-AAD (eBioscience, San Diego, CA) according to eBioscience’s Best Protocols. The cells were assessed with a fluorescence activated cell sorter (FACS) Aria II (BD Biosciences, San Diego, CA) and analyzed using FCS Express 4 Plus (De Novo Software, Los Angeles, CA).

### Immunoblots and enzyme-linked immunosorbent assay (ELISA)

To measure the cytokine secretion of the infiltrated CD4+ T cells, lymphocytes were sterilely isolated from allografts using a CD4+ T cell isolation kit (Miltenyi Biotec, Bergisch Gladbach, Germany) and cultured in neutral stimulate with plate-bound anti-CD3 (5 µm/ml, eBioscience) and anti-CD28 (10 µm/ml, eBioscience) antibodies. The supernatants were removed after 48 h, and cytokine levels were analyzed using a mouse cytokine antibody array (RayBiotech Inc., Norcross, GA).

### Quantitative real-time PCR

Total RNA was extracted from allografts or cultured cells using TRIzol Reagent (Invitrogen, Carlsbad, CA) following the manufacturer’s instructions. cDNA was prepared using the PrimeScript RT Master Mix reverse transcription kit (TaKaRa, Shiga, Japan). The PCR mixture was prepared using SYBR Premix EX Taq (TaKaRa, Shiga, Japan), and transcription levels were detected using a BioRad CFX96 (Bio-Rad Laboratories Inc., Hercules, CA). The relative expression levels were normalized to the expression of GAPDH using the ΔΔCt method. The primers used were as follows: T-bet, forward: 5′-CAACAACCCCTTTGCGAAAG-3′, reverse: 5′-TCCCCCAAGCAGTTGACAGT-3′; GATA-3, forward: 5′-AGCCACATCTCTCCCTTCAG-3′, reverse: 5′-AGGGCTCTGCCTCTCTAACC-3′; RORγt, forward: 5′-TGCAAGACTCATCGACAAGG-3′, reverse: 5′-AGGGGATTCAACATCAGTGC-3′; Foxp3, forward: 5′-ACTGGGGTCTTCTCCCTCAA-3′, reverse: 5′-GTGGGAAGGTGCAGAGTAG-3′; iNOS, forward: 5′-GTTCTCAGCCCAACAATACAAGA-3′, reverse: 5′-GTGGACGGGTCGATGTCA -3′; Arg1, forward: 5′-CTCCAAGCCAAAGTCCTTAGAG-3′, reverse: 5′-GGAGCTGTCATTAGGGACATC-3′; Mrc1, forward: 5′-AATGAAGATCACAAGCGCTGC-3′, reverse: 5′-TGACACCCAGCGGAATTTCT-3′, GAPDH, forward: 5′-TTCACCACCATGGAGAAGGC-3′, reverse: 5′-GGCATGGACTGTGGTCATGA-3′.

### Cell culture and monocyte/Treg coculture

Mouse splenic CD4+ T cells were purified with a CD4+ T cell isolation kit (Miltenyi Biotec, Bergisch Gladbach, Germany) and stimulated with plate-bound anti-CD3 (5 µm/ml, eBioscience) and anti-CD28 (10 µm/ml, eBioscience) antibodies together with IL-12 (10 ng/ml) for Th1 differentiation, IL-4 (10 ng/ml) for Th2 differentiation, TGF-β (5 ng/ml) and IL-6 (20 ng/ml) for Th17 differentiation, and TGF-β (5 ng/ml) alone for Treg differentiation. All cytokines were obtained from eBioscience. Monocytes and Treg were cocultured at a ratio of 2∶1 according to a previously reported method [21]. Treg and monocytes were isolated from spleen using a CD4+CD25+ isolation kit or CD11b+ isolation kit (Miltenyi Biotec, Bergisch Gladbach, Germany). The purity of CD11b+ isolated monocytes is greater than 88% by flow cytometry. Cells were cocultured with RPMI 1640 and stimulated by anti-CD3 (5 µm/ml, eBioscience) and LPS (50 ng/ml, Sigma, St Louis, USA) for 36 h.

### Statistical analysis

The data are expressed as the mean±SD, and comparisons between groups were conducted using unpaired Student’s *t* tests. The allograft survival curve was plotted using the Kaplan-Meier method, and differences were determined using a log-rank test. All data were analyzed using Prism 5.0 (GraphPad Software, La Jolla, CA). P values less than 0.05 were considered statistically significant.

## Results

### PPARγ-deficient T cells exhibit significant decreases in cardiac allografts survival and aggravate chronic allograft rejection

**Figure 1:**
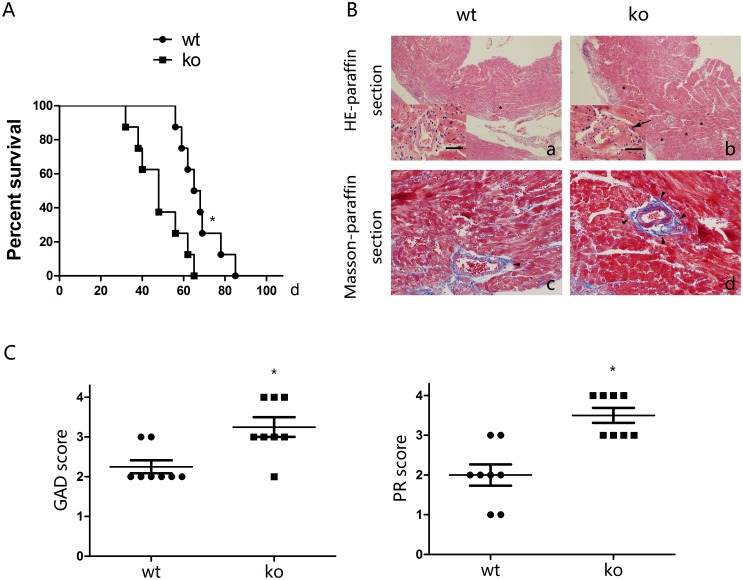
PPARγ-deficient T cells reduce survival and augment lesions associated with chronic rejection. A. The survival time of cardiac allografts in T cell-PPARγ^ko^ mice was significantly shorter than that of reduced compared with WT littermates. B. Cardiac allograft (bm12) sections of lesions 40 days after transplantation from T cell-PPARγ^ko^ mice (ko) or WT littermates. (a, b) HE staining, major view magnification 40×, left bottom view magnification 400×. Scale bars = 100 µm. (c, d) Masson trichrome staining, magnification 400×. T cell-PPARγ^ko^ mice exhibited severe lesions. Arrows indicate obvious intimal hyperplasia, asterisks indicate leukocytic infiltration, and filled arrowheads indicate collagen deposition. C. Graft coronary artery disease (GAD) and parenchymal rejection (PR) scores in T cell-PPARγ^ko^ mice were considerably increased compared with WT littermates. The data are presented as the mean±SD for each group (n = 8), *p<0.05.

We established a model of heterotrophic cardiac transplantation using an MHC class II - mismatched model. To investigate the contribution of PPARγ expression in T cells to chronic allograft rejection, we used T cell-PPARγ^ko^ mice as recipients. We evaluated allografts daily by palpation to detect their function. In WT littermates, a median allograft survival (MST) of 67 days was observed after transplantation; however, the MST in T cell-PPARγ^ko^ mice was only 48 days (Figure 1A). To detect lesions of chronic allograft rejection, HE and Masson staining were performed on the allografts 40 days after transplantation. In WT littermates, perivascular leukocytic infiltration, CAV and destruction of the cardiac muscle structure were observed, however, in T cell-PPARγ^ko^ mice, more intensive chronic allograft rejection signs were noted compared with the WT littermates. (Figure 1B). Consistent with these observations, the GAD and PR scores of T cell-PPARγ^ko^ mice were increased compared with WT littermates 40 days after transplantation (Figure 1C). These results suggested that recipients with PPARγ-deficient T cells exhibited decreased allograft survival and aggravated lesions of chronic allograft rejection.

### T cell PPARγ deficiency increases CD4+ T cell infiltration in cardiac allografts

**Figure 2:**
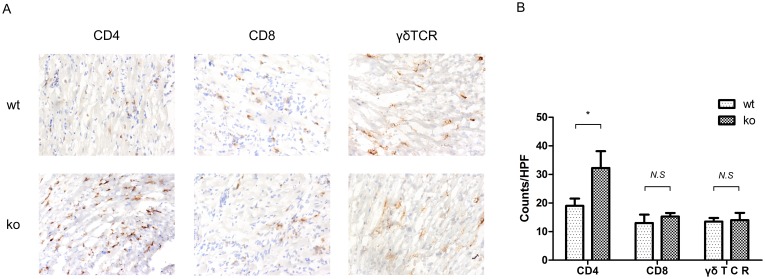
Infiltration CD4+, CD8+, and γδTCR+ cells in donor hearts from T cell-PPARγ^ko^ mice or WT littermates. A. Immunohistochemical staining of CD4+, CD8+, and γδTCR+ cells in allografts 40 days after transplantation. Top, allograft from T cell-PPARγ^ko^ mice (ko); bottom, allograft from WT littermates (wt). B. Quantitative analysis of CD4+, CD8+, and γδTCR+ cells in allografts counted per high power field (HPF). The data are presented as the mean±SD of 8 fields per group, *p<0.05.

Leukocyte infiltration into allografts is a hallmark and pathogenic factor of chronic allograft rejection. Given that we identified more severe lesions in T cell-PPARγ^ko^ mice, we next detected the infiltration of T cell subsets into the allograft 40 days after transplantation. The CD4+, CD8+, and γδTCR+ T cell subsets in allografts were counted per high-power field (HPF) using immunohistochemical staining. Compared with the T cell subsets in the cardiac allografts of WT littermate recipients, a remarkable increase in infiltrated CD4+ T cells was observed in T cell-PPARγ^ko^ mice. In contrast, the numbers of CD8+ and γδTCR+ T cells did not differ between the groups (Figure 2A, B). As T cell-PPARγ^ko^ mice found to have CD4+, CD8+, γδTCR+ T cells and CD11b+ monocytes ratio comparable to those of C57BL/6 and WT littermate initiate before operation (Figure S1).

### PPARγ-deficient T cells alter cytokine secretion and the polarization of CD4+ T cells in cardiac allografts

**Figure 3:**
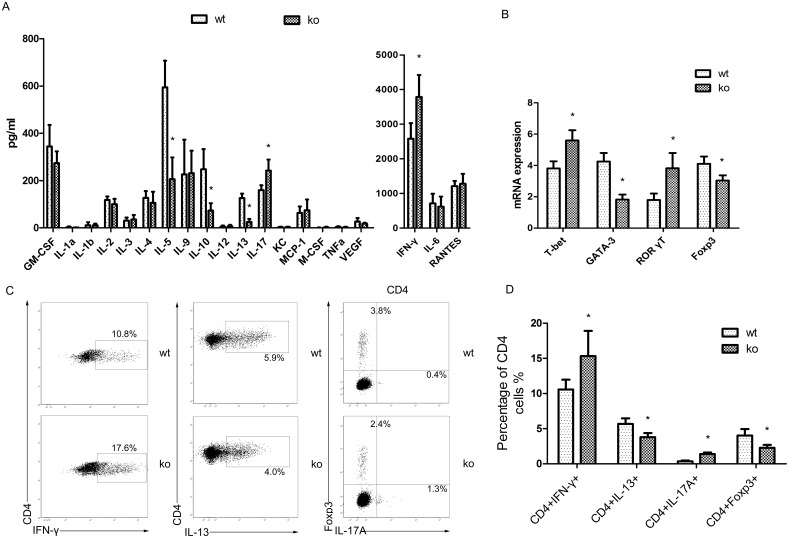
Cytokines and transcription factor expression in infiltrating CD4+ T cells in cardiac allografts. A. Analysis of cytokine secretion by infiltrating CD4+ T cells from T cell-PPARγ^ko^ mice (ko) and WT littermates (wt) using a cytokine antibody array 40 days after transplantation. B. The transcription levels of the transcription factors T-bet, GATA-3, RORγt, and Foxp3 in allografts were measured by RT-PCR 40 days after transplantation. C. Graft-infiltrating CD4+ T cells were isolated 40 days after transplantation. These cells were stained with CD45, CD4, and 7-AAD then intracellularly stained with IFN-γ, IL-13, IL-17A, and Foxp3 for assessment by flow cytometry. D. The percentage of CD4+IFN-γ+/CD4+IL-13+/CD4+IL-17A/CD4+Foxp3+ cells in infiltrating CD4+ T cells was determined by flow cytometry. The data are presented as the mean±SD for each group (In panels A and B n = 10; in panels C and D n = 5), *p<0.05.

Most studies have focused on Th1 (secreting IFN-γ), Th2 (secreting IL-4/13) and Th17 (secreting IL-17) cells, which were considered the predominant contributors to chronic allograft rejection. Because we found increased CD4+ T cell infiltration in PPARγ-deficient T cell recipients in a previous study, we used a cytokine antibody array to detect cytokine secretion by infiltrated CD4+ T cells *in vitro* under the neutral stimulation conditions with anti-CD3/28 40 days after transplantation. We confirmed that in PPARγ-deficient T cell recipients, CD4+ T cells secreted higher levels of Th1-related IFN-γ and Th17-related IL-17. In contrast, Th2-related IL-5, IL-10 and IL-13 cytokine secretion decreased compared with WT littermates (Figure 3A). We also used RT-PCR to measure the mRNA levels of transcription factors specific for CD4+ T cells in the cardiac allografts. The cardiac allografts from PPARγ-deficient T cell recipients expressed higher levels of Th1- and Th17- related T-bet and RORγt and reduced levels of Th2- and Treg- related GATA-3 and Foxp3 (Figure 3B). The flow cytometry data on the infiltrated CD4+ T cells in the allograft revealed a variation trend consistent with the RT-PCR data (Figure 3C, D).

### PPARγ-deficient CD4+ T cells enhance Th1 and Th17 polarization *in vitro*

**Figure 4:**
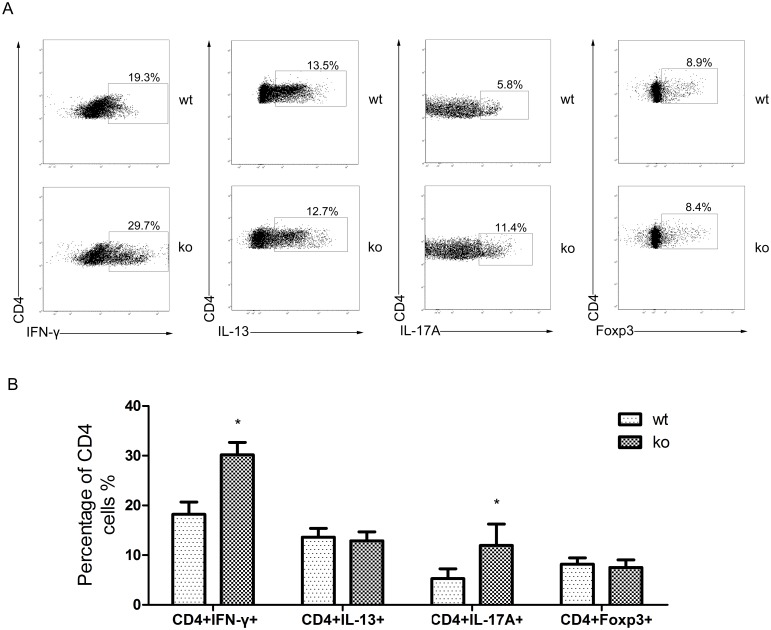
T cell-specific-PPARγ knockout changes CD4+ T cell subsets differentiation. A. CD4+ T cells were isolated from T cell-PPARγ^ko^ mice (ko) and WT littermates (wt) spleens and then cultured in protocols that induce T cell polarization into Th1, Th2, Th17, and Treg. After 4 days of culture, these cells were assessed by flow cytometry. B. The percentage of CD4+IFN-γ+/CD4+IL-13+/CD4+IL-17A/CD4+Foxp3+ cells in each cultured group of total CD4+ cells. The data are presented as the mean±SD for each group (n = 5), *p<0.05.

Previous experiments indicated that CD4+ T cell subsets varied in PPARγ-deficient T cell allografts. We tested whether the PPARγ-deficient T cells exhibited altered polarization into Th1, Th2, Th17, and Treg cells. We isolated CD4+ T cells from the spleens of T cell-PPARγ^ko^ mice or WT littermates and cultured them under Th1/Th2/Th17/Treg-polarizing conditions for 4 days. Cells were then harvested, and intracellular cytokine levels were detected using flow cytometry with intracellular staining for IFN-γ, IL-13, IL-17A, and Foxp3. A remarkable increase in IFN-γ and IL-17A production was observed in the PPARγ-deficient CD4+ T cells. However, the percentage of CD4+IL-13+ and CD4+Foxp3+ T cells did not change (Figure 4A, B). These findings suggested increased differentiation into the Th1 and Th17 subsets in PPARγ-deficient CD4+ T cells. The decreased Th2 and Treg subsets in the allografts were likely attributed to the increase in Th1 and Th17 cells and not to a failure to differentiate.

### The effect of T cell PPARγ deficiency on macrophage polarization *in vivo* and *in vitro*

**Figure 5:**
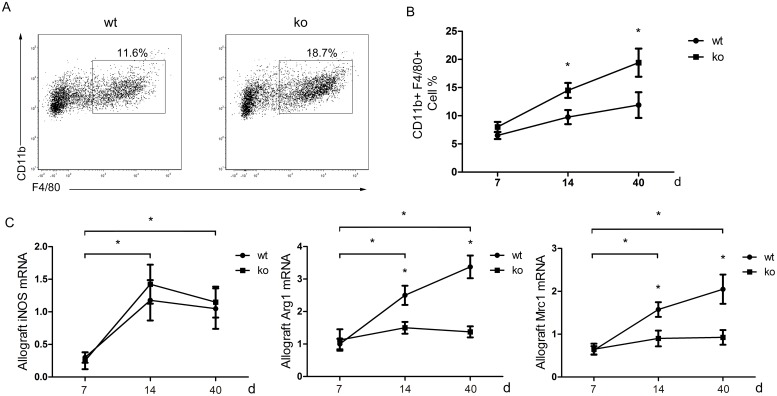
Analysis of macrophage infiltration and polarization in cardiac allografts. A. Flow cytometry analyses of the proportion of CD11b+ F4/80+ macrophages infiltrating cardiac allografts 40 days after transplantation. B. Dynamic analyses of the proportion of infiltrated macrophages in the total mononuclear cells in allografts at 7, 14, and 40 days after transplantation. C. iNOS, Arg1 and Mrc1 transcription levels in allografts were measured by RT-PCR at 7, 14 and 40 days after transplantation. The data are presented as the mean±SD for each group (n = 5), *p<0.05.

**Figure 6:**
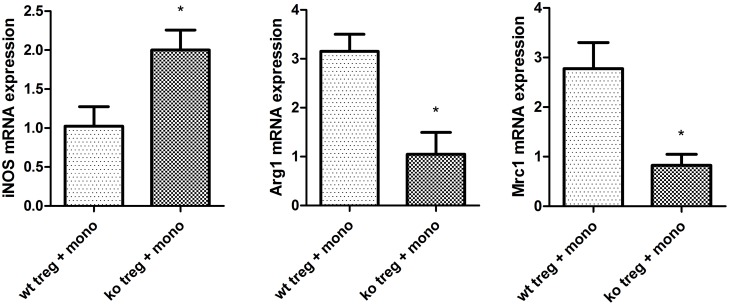
PPARγ^ko^ Treg fail to induce AAM in a monocyte and T cell coculture. CD4+CD25+ T cells were isolated from the spleens of T cell-PPARγ^ko^ mice (ko) and WT littermates (wt) and cocultured with CD11b+ monocytes from PBMC C57BL/6 mice. The transcription level of iNOS, Arg1, and Mrc1 was assessed in monocytes after 48 h of coculture by RT-PCR. The data are presented as the mean±SD for each group (n = 5), *p<0.05.

Previous studies have suggested that macrophages participate in chronic allograft rejection and that T cell subsets may influence macrophage polarization [9], [10], [21]. Therefore, we next examined whether PPARγ-deficient T cells affect macrophage infiltration and polarization in cardiac allografts. Flow cytometry was performed to detect the amount of CD11b+ F4/80+ macrophage infiltration at 7, 14, and 40 days after transplantation. In the cardiac allografts from T cell-PPARγ^ko^ mice, the number of macrophages was significantly increased compared with the WT littermates allografts (Figure 5A, B). To identify macrophage polarization into CAM and AAM, we measured the expression of CAM-related-iNOS, AAM-related-Arg1, and Mrc1 using RT-PCR at 7, 14, and 40 days after transplantation. Arg1 and Mrc1 mRNA levels did not increase in T cell-PPARγ^ko^ mice, whereas the levels were significantly elevated in WT littermates on days 14 and 40 post-grafting. In addition, no significant differences in the mRNA levels of CAM-related-iNOS were noted in the two groups. Therefore, in T cell-PPARγ^ko^ recipients mice, macrophages failed to differentiate into AAM in local allografts. Because some studies have demonstrated that Treg can induce AAM polarization, we hypothesized that the PPARγ-deficient Treg failed to induce AAM polarization. We isolated autologous CD4+CD25+ Treg from the spleens of T cell-PPARγ^ko^ mice or their WT littermates and cocultured these cells with CD11b monocytes from C57BL/6 mice. After 2 days, cells were collected for RT-PCR detection. Arg1 and Mrc1 transcription levels were reduced in PPARγ-deficient Treg cocultured with monocytes (Figure 6). All of the above results demonstrated that PPARγ-deficient T cells impact AAM polarization *in vivo* and *in vitro*.

### PPARγ-deficient T cells abrogate the protective effect of a PPARγ agonist

**Figure 7:**
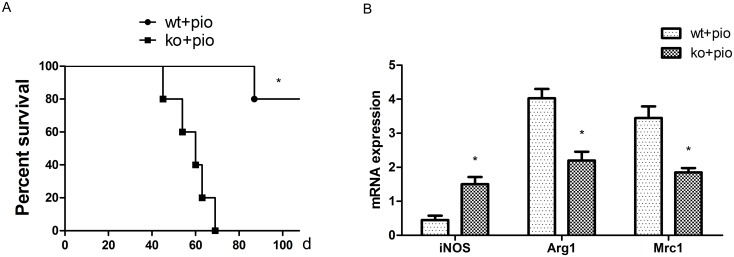
The beneficial effects of pioglitazone on the survival of cardiac allografts were counteracted in T cell-PPARγ^ko^. A. T cell-PPARγ^ko^ mice (ko) abrogate the protective effect of pioglitazone on the survival curve. B. iNOS, Arg1, and Mrc1 transcription levels in allografts were measured by RT-PCR 40 days after transplantation. The data are presented as the mean±SD for each group (n = 5), *p<0.05.

In our prior study, we demonstrated that PPARγ-deficient T cells reduce the polarization of AAM. Other researchers have observed that PPARγ agonists enhance AAM differentiation [42]. We examined whether PPARγ-deficient T cells neutralize the protective effect of PPARγ agonists in chronic allograft rejection. After treatment with the PPARγ agonist pioglitazone, the median allograft survival significantly decreased in T cell-PPARγ^ko^ mice (Figure 7A). Moreover, Arg1 and Mrc1 transcription levels in allografts significantly decreased in T cell-PPARγ^ko^ groups compared with WT littermates administered pioglitazone 40 days after transplantation (Figure 7B).

## Discussion

Several studies have demonstrated the protective role of PPARγ agonists in chronic allograft rejection [15], [22], [23]. In these studies, the allogeneic reactions of cell infiltration and proinflammatory cytokine secretion are suppressed by PPARγ agonists. However, the details regarding how PPARγ influences the immune response in chronic allograft rejection are unknown. Recent studies have reported that PPARγ plays an important immunoregulatory function in autoimmune diseases, such as experimental autoimmune encephalomyelitis (EAE) [24], allergic airway inflammation [25], trinitrobenzene sulfonic acid-induced colitis [26], and insulin resistance [27]. In these disease models, PPARγ is the key factor promoting the differentiation of CD4+ T cells and macrophages. Our previous study found that PPARγ activation with eicosapentaenoic acid can impact the balance between Th17/Treg cells and protect cardiac allografts [14], consistent with its effect on autoimmune diseases. Therefore, we hypothesize that PPARγ controls T cell subset polarization in chronic allograft rejection and influences tolerance to allografts after transplantation.

In this study, we established a MHC class II-mismatched chronic allograft rejection model. To eliminate confusion due to non-selective PPARγ agonists in the entire immune system, we used B6 background PPARγ fl/fl; Lck-Cre^+^ T cell-specific PPARγ knockout mice and WT littermates as recipients. In the T cell-PPARγ^ko^ group, leukocyte infiltration and CAV were aggravated, and allograft survival decreased compared with WT littermates. These results confirmed that although PPARγ influences both T cells and macrophages [28], in chronic allograft rejection, T cells expressing PPARγ may participate in the primary function of transplant tolerance. Previous studies have suggested that CD4+, CD8+, and γδTCR+ T cells infiltrated into the allografts and contributed to allograft rejection [19], [29]. We used immunohistochemical staining to detect infiltrated T cells in cardiac allografts 40 days after transplantation. The number of infiltrated CD4+ T cells was significantly increased in allografts in T cell-PPARγ^ko^ recipients. This result suggested that the infiltration of CD4+ T cells in allografts increased with PPARγ knockout and caused the intensive lesion.

CD4+ T cells and their secretion of various cytokines participate in allogeneic rejection [30]. We isolated infiltrated CD4+ T cells in allografts and examined their secreted cytokines. Based on RT-PCR and flow cytometry, the percentage of Th1 and Th17 increased, whereas Th2 and Treg decreased in the T cell-PPARγ^ko^ group. These results implied that PPARγ knockout altered the subsets of CD4+ T cells. We next examined T cell differentiation *in vitro*. In contrast to the allografts, Th1 and Th17 differentiated more efficiently when PPARγ was knocked out, whereas Th2 and Treg differentiation remained unchanged. These results are consistent with those of previous studies that also used conditional PPARγ knockout mice cells [24], [26]. Other laboratories using PPARγ agonists reached similar conclusions. Augstein et al. used the PPARγ agonist troglitazone to reduce T cell production of IFN-γ in an autoimmune diabetes model [31]. In an allergic airway inflammation model, a PPARγ agonist decreased NF-κB activity and reduced IL-17 release [25]. We concluded that the number of Th1 and Th17 cells infiltrated into allografts increased remarkably and led to a relative decrease in Th2 and Treg cells. Th1 is a classic proinflammatory cell and has been shown to be principally responsible for chronic allograft rejection [32]. Yuan et al. demonstrated that Th17 secretion of the proinflammatory cytokine IL-17A resulted in CAV when T-bet was knocked out [6]. Th2 and Treg cells have always been recognized as elements that prolong allograft survival [33], [34] and were present at a lower ratio in allografts than in WT littermates. Their protective effect was limited with PPARγ deficiency.

Recent studies have reported that macrophages are a major component in the immune response involved in allograft rejection [35], [36]. CAM accelerate the immune response, whereas AAM have repair and anti-inflammatory abilities [37]. Moreover, PPARγ agonists skewed monocytes toward AAM polarization [13]. We have observed that without PPARγ in T cells, PPARγ agonists cannot exert protective function on macrophages. In additional studies, we investigated macrophage polarization in T cell-PPARγ^ko^ mice. Previous studies have indicated that IL-4 and IL-13 binding to IL-4Rα and IL-13Rα1 resulted in downstream phosphorylation of STAT6, thereby causing macrophage polarization [38]–[40]. More recently, Szanto et al. proposed that PPARγ plays a critical role in these path ways because IL-4 signaling activates PPARγ through STAT6 interactions with PPARγ and promotes their binding to PPRE [41]. Indeed, the IL-4/13-STAT6-KLF4-PPARγ axis is regarded as an essential regulator of CAM/AAM polarization and function [42]. In our experiments, the ratio of Th2 decreased, and Th2-related cytokine production was reduced. The AAM markers Arg1 and Mrc1 were down-regulated after T cell specific knockout of PPARγ. Additionally, Tiemessen et al indicated that Treg have the ability to induce AAM *in vitro* [21]. Thus, we employed the same method to demonstrate that PPARγ-deficient Treg failed to induce AAM polarization. The reduced Th2-related cytokine release together with the elimination of the ability of Treg to induce AAMs indicated that the decline in number of AAMs in PPARγ-deficient allografts might be the cause of the severity of chronic rejection. To confirm that T cells influenced by PPARγ were the primary cause of AAM polarization in transplantation tolerance, we administered a PPARγ agonist to T-cell-PPARγ^ko^ mice and WT littermate recipients. Indeed, PPARγ-deficient T cells significantly reduced the survival of allografts and suppressed AAM polarization in the pioglitazone-treated groups.

In conclusion, the present study demonstrated that PPARγ expression is important for maintaining subsets of CD4+ T cells and macrophages in transplantation tolerance. The protective effect of a PPARγ agonist in allograft rejection potentially functions through these immune processes. However, one of the remarkable features in chronic rejection is CAV, whose pathologic lesions occasionally appear similar to atherosclerosis but occur through a unique mechanism [43]. Vladimir et al. reported that macrophage-specific PPARγ knockout increased atherosclerosis [44]. PPARγ expression in macrophages requires further investigation. Although PPARγ agonists have effective immunoregulatory capacity in many autoimmune diseases and transplant rejection animal models, the side effects of conventional thiazolidinedione drugs (TZD) have been reported in recent clinical studies [12]. Because the mechanism of the protective effect of PPARγ in chronic rejection has been demonstrated, we expect that PPARγ-targeted therapeutics might serve as a novel method to promote transplant tolerance.

## Supporting Information

Figure S1 **T cell-PPARγ^ko^ mice have normal T cell and monocyte subpopulations.** Flow cytometry analyzing the proportion of T cell and monocyte subpopulations from T cell-PPARγ^ko^ mice are normally comparable to those of C57BL/6 mice and WT littermate before operation in the spleens. The data are presented as the mean±SD for each group (n = 5).
(TIF)
