# Supplementary figures and images for: Peroxisome Proliferator-Activated Receptor γ Deficiency in T Cells Accelerates Chronic Rejection by Influencing the Differentiation of CD4+ T Cells and Alternatively Activated Macrophages
Source: PLoS One. 2014 Nov 10;9(11):e112953. doi: 10.1371/journal.pone.0112953 (PMC4226585; doi:10.1371/journal.pone.0112953)

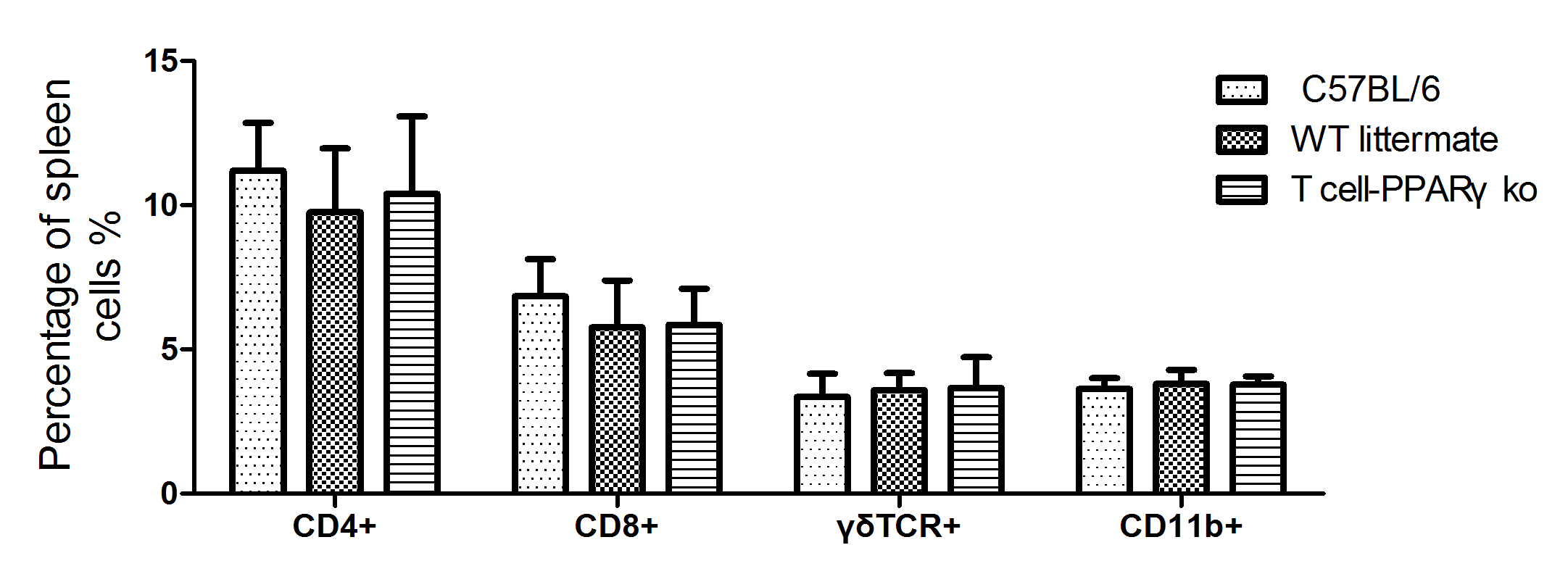

Supplement: Figure S1 — T cell-PPARγko mice have normal T cell and monocyte subpopulations. Flow cytometry analyzing the proportion of T cell and monocyte subpopulations from T cell-PPARγko mice are normally comparable to those of C57BL/6 mice and WT littermate before operation in the spleens. The data are presented as the mean±SD for each group (n = 5). (TIF) [file pone.0112953.s001.tif]
